# An Electrochemical Immunosensor for Sensitive Detection of the Tumor Marker Carcinoembryonic Antigen (CEA) Based on Three-Dimensional Porous Nanoplatinum/Graphene

**DOI:** 10.3390/mi11070660

**Published:** 2020-07-03

**Authors:** Aihua Jing, Qiong Xu, Wenpo Feng, Gaofeng Liang

**Affiliations:** 1 School of Medical Technology and Engineering, Henan University of Science and Technology, Luoyang 471023, China; aihuaj@haust.edu.cn (A.J.); xuqiong@stu.haust.edu.cn (Q.X.); fwp238@haust.edu.cn (W.F.); 2 Medical College, Henan University of Science and Technology, Luoyang 471023, China

**Keywords:** carcinoembryonic antigen (CEA), three-dimensional porous graphene, platinum (Pt), electrochemical immunosensor, tumor marker, detection

## Abstract

Carcinoembryonic antigen (CEA) is an important broad-spectrum tumor marker. The quantitative detection of a low concentration of CEA has important medical significance. In this study, three-dimensional porous graphene-oxide-supported platinum metal nanoparticles (3DPt/HGO) composites were prepared by a wet chemical method and modified on an electrode with enhanced conductivity, a large surface area, and good adsorption of immobilizing antibodies (Ab_1_). Horseradish peroxidase (HRP)-functionalized Au nanoparticles were fabricated to label the secondary antibodies (Ab_2_). The proposed immunosensor showed a good linear relationship in the range of 0.001–150 ng/mL for CEA and a detection limit of 0.0006 ng/mL. The immunosensor had high sensitivity, good stability and reproducibility, and has great application prospects for the clinical diagnosis of cancer.

## 1. Introduction

Carcinoembryonic antigen (CEA), an important broad-spectrum tumor biomarker, is used as an efficient prognostic indicator in the early clinical diagnosis and disease prevention of pancreatic cancer [1,2], colorectal cancer [3,4], breast cancer [5,6], lung cancer [7,8], and gastric cancer [9,10,11]. The sensitive detection of low levels of CEA in serum is of great clinical significance in assessing tumor status and therapeutic efficiency, as well as in the early clinical diagnosis of tumor. To date, different strategies have been used to measure CEA, such as radioimmunoassay [12], chemiluminescence analysis [13], enzyme-linked immunosorbent assay [14,15], and electrochemistry [16,17]. Among these methods, electrochemical immunosensors [18,19] have attracted considerable attention due to their distinct advantages, such as high selectivity, fast response, low sample requirements, miniaturization, and excellent sensitivity [20,21,22,23]. Nonetheless, new immunosensors fabricated with nanomaterials that have excellent performance for the quick and simple detection of CEA with high sensitivity and selectivity are still urgently needed in the medical field.

The signal amplification system is vital to improving detection sensitivity and selectivity. Generally, it contains two parts: a platform and a label. The platform of the designed immunosensor is the first key part in the quantitative assay of CEA. It should have a good capacity to immobilize more antibodies and enhance the electron transfer. Therefore, various nanomaterials with good electron transfer ability, biocompatibility, and large specific surface area are suitable for the immobilization [24]. Three-dimensional graphene interfacial materials have been widely used as supporting materials in electrochemical biosensors because the good electrical conductivity [25], thermal conductivity [26], and biocompatibility [27] of graphene can enable fast electron transfer and rapid mass transport with large surface areas [28]. Typically, graphene oxide with nanohole macrostructures exhibits improved electron transfer dynamics compared to those of non-holey graphene oxide [29]. Three-dimensional porous graphene structures serving as electrode materials are expected to show improved electron transfer and mass transport during the electrochemical detection of tumor markers. Furthermore, anchoring nanoscale noble metal particles onto specific supports such as 3D graphene can improve more highly active centers on the 3D noble metal/graphene composite [30]. Pt nanoparticles have been used as the substrate material for the incubation of the capture antibody (Ab_1_) in immunosensors. Pt nanoparticles can aggregate due to their small particle size and large electrochemical active area. They were decorated on a support material with high surface area and high electrochemical stability to keep their activity [31]. Pt nanoparticles functionalized with various graphene structures, for example, porous Pt/ionic liquid/graphene [32], PtPd/nitrogen/graphene [33], NiAuPt/graphene [22], porous platinum/PdPt nanocages [34], and gold/bienzyme/platinum/graphene [35] have been immobilized on electrodes to accelerate the electron transfer and provide a superior microenvironment for capturing more antibody (Ab_1_), thereby improving the sensitivity of the CEA immunosensors. There is still great interest in and need for developing new 3D graphene and Pt nanoparticle composites. Here, three-dimensional porous graphene-oxide-supported platinum metal nanoparticles (3DPt/HGO) composites, with enhanced conductivity and large surface areas, are modified on an electrode in order to immobilize Ab_1_ and improve the sensitivity of the electrochemical immunosensor.

The label of the designed immunosensor is the second key part of the quantitative assay of CEA. It should have good catalytic activity itself or can act as an electroactive substance in detection [21]. Noble metal nanoparticles often work as electroactive substances in electrochemical immunosensors due to their good biocompatibility, high anti-interference capability and excellent electrical conductivity [36]. Here gold nanoparticles were used in this immunosensor to provide a suitable interface to anchor more Ab_2_ to amplify electrochemical signals. 

**Figure 1:**
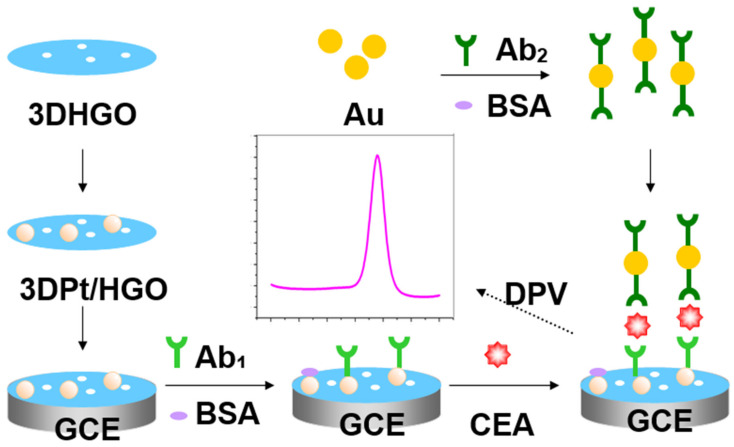
The schematic illustration of the fabrication process of the carcinoembryonic antigen (CEA) immunosensor. Ab_1_: capture antibody; Ab_2_: secondary antibody; 3DPt/HGO: 3D porous graphene-oxide-supported platinum metal nanoparticles; GCE: glass carbon electrode.

In this work, a sandwich-type electrochemical immunosensor comprised of a 3D composite of Pt nanoparticles deposited on 3D graphene oxide with nanopores was designed for the electrochemical detection of the tumor biomarker CEA. Figure 1 displays a graphical illustration of the fabrication and detection process of the sandwich-type immunoassay. 3DPt/HGO with high conductivity and large active surface area was used as the substrate material of the immunosensor for the capture of Ab_1_. Gold nanoparticles were used as signal tags to improve the sensitivity of the immunosensor by capturing the secondary antibody (Ab_2_)/HRP (horseradish peroxidase). The signal of the proposed immunosensor was linear from 0.001 to 150 ng/mL CEA and had a low detection limit of 0.0006 ng/mL. The developed immunosensor was tested in human serum samples for CEA detection. The obtained satisfactory recovery of the ultrasensitive immunosensor indicated a potential practical application in clinical diagnostics.

## 2. Experimental Sections

### 2.1. Instruments and Reagents

A JEOL JSM-7800F field-emission scanning electron microscope (FE-SEM) was used to characterize the morphologies and energy-dispersive spectrometry (EDS) of the product. CEA, alpha-fetoprotein (AFP), HRP-conjugated rabbit anti-mouse IgG, bovine serum albumin (BSA), and chloroplatinic acid were purchased from Shanghai Sangon Bioengineering Co. (Shanghai, China). The mouse-derived CEA antibody was purchased from Linc-Bio Science Co. Ltd. (Shanghai, China). L-Cysteine, glucose, vitamin C, and L-glutamine were purchased from Tianjin Fengchuan Chemical Reagent Technology Co. (Tianjin, China). Phosphate buffer solutions (PBS, pH 7.4) were made from 0.1 M Na_2_HPO_4_ and 0.1 M KH_2_PO_4_ solutions. All chemicals and solvents used were of analytical grade. Double-distilled water was used. The human serum was collected and treated as follows: the blood was extracted and put into a centrifuge tube, then centrifuged for 5 min at 1000 rpm at low temperature (4 °C). The supernatant (serum) was carefully taken out and frozen for future use.

### 2.2. Preparation of 3D Porous Graphene Oxide (3DHGO)

The GO and HGO were prepared by a modified Hummer’s method, as reported by our group [27]. The 3DHGO was prepared by a hydrothermal method. Briefly, 10 mL of HGO solution (2 mg/mL) was sealed and maintained at 180 °C for half a day. Then, at room temperature, HI (55%) was added and heated at 100 °C for 3 h. After that, the obtained results were dialyzed in ultrapure water for 2 days [37].

### 2.3. Preparation of 3DPt/HGO Composites

The 3DPt/HGO composites were prepared by a modified wet chemical method. Typically, 0.5 mg/mL of 3DHGO was first thoroughly ultrasonicated for 30 min. Then, 9.2 mL of 10 mM H_2_PtCl_6_·6H_2_O was added to the above 3DHGO suspension at 0 °C and continuously stirred for 30 min. The reaction mixture was further purified by centrifugation at 12,000 rpm for 30 min and washed 3-4 times with deionized water. Finally, the solution of 3DPt/HGO nanocomposites was obtained [38].

### 2.4. Preparation of Ab_2_-HRP/Au Bioconjugate

The gold nanoparticles were first synthesized according to a published method [39]. The solutions of gold nanoparticles (2.0 mg/mL, 1 mL) and Ab_2_ (10 μg/mL, 1 mL) were well-mixed and stirred for 24 h at 4 °C. Then, 0.1% BSA was added to block the remaining active sites of the gold nanoparticles. After centrifugation, the resulting Ab_2_-HRP/Au bioconjugate was dispersed in 1 mL PBS solution and stored at 4 °C.

### 2.5. Fabrication of the Electrochemical Immunosensor

Figure 1 shows a schematic fabrication of the electrochemical immunosensor. A glass carbon electrode (GCE, diameter 3 mm) was polished with 0.3- and 0.05-μm alumina slurry and cleaned by an ultrasonic cleaner with HNO_3_ (1:1), acetone, anhydrous ethanol, and secondary distilled water. The clear GCE was dried at room temperature. Following this, 10 μL of 3DPt/HGO nanocomposite was dropped on the GCE surface, and then 5 μL of anti-CEA was dropped evenly on the 3DPt/HGO nanocomposite surface. The electrode was cleaned with pH 7.4 PBS and incubated in a refrigerator for 13 h. After this, the 3DPt/HGO was immersed in 1.0 wt% BSA solution for 35 min at 37 °C to block the remaining active sites in order to avoid non-specific adsorption. Then, the immunosensor was cleaned with PBS and dried using a high-purity nitrogen steam. Subsequently, the sensor was incubated in solutions with various concentrations of CEA for 40 min at 37 °C, washed with PBS solution, and dried under a steam of nitrogen. Finally, the immunosensor was immersed in Ab_2_-HRP/Au solution prepared as in Section 2.4. for the sandwich immunoreaction. After cleaning with 0.01 M PBS, the immunosensor was dried using nitrogen gas.

### 2.6. Electrochemical Detection

A CHI660E electrochemical workstation (Shanghai Chenhua Instrument Co., Ltd, Shanghai, China) recorded all the electrochemical detections with a three-electrode system containing a platinum auxiliary wire, a modified GCE, and a saturated calomel reference. The solutions were degassed with nitrogen to remove O_2_. Cyclic voltammetry (CV), differential pulse voltammetry (DPV), and electrochemical impedance spectroscopy (EIS) were carried out in the solution (0.1 M PBS (pH 7.4) + 1 mM Fe(CN)_6_^3−^/^4−^ (1:1) + 0.1 M KCl).

## 3. Results and Discussion

### 3.1. Characterization of Materials

**Figure 2:**
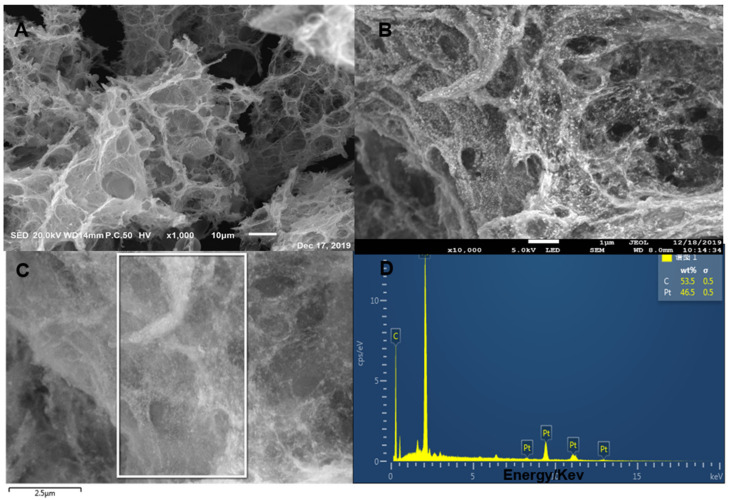
Scanning electron microscope (SEM) images at low (**A**) and high (**B**) magnifications of 3DPt/HGO composite, and an SEM image (**C**) with the corresponding energy-dispersive spectrometry (EDS) (**D**).

Figure 2 shows the SEM of 3D Pt/HGO composites with different magnifications and the corresponding EDS. From the SEM images of Figure 2A, it can be seen that 3DHGO had a dense pore structure, and the pore size was mainly between the nanometer and micron size. Figure 2B shows that Pt nanoparticles grew uniformly on 3DHGO; the size was uniform and the particles were small. More importantly, the dispersion of Pt particles was more uniform and there was little obvious agglomeration. The formation of Pt nanoparticles on the surface of HGOs might be due to the electrostatic interactions of the delocalization π-π bonds between graphenes and the charged Pt nanoparticles, indicating the successful assembly of Pt nanoparticles on HGOs [40]. The main co-existing elements including C and Pt were obvious for the 3DPt/HGO composite (Figure 2D).

### 3.2. Electrochemical Behavior of Modified Electrodes

**Figure 3:**
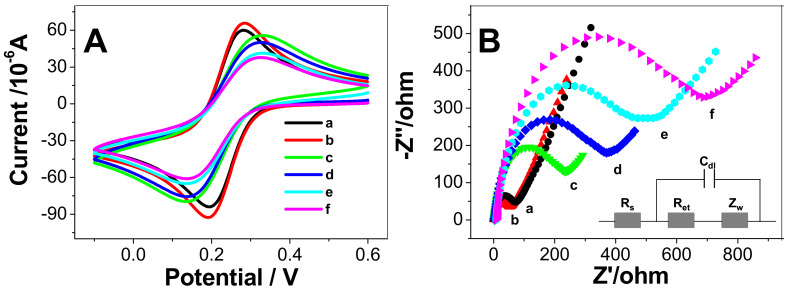
(**A**) Cyclic voltammetry (CVs) and (**B**) Nyquist plots of bare GCE (a), 3DPt/HGO/GCE (b), Ab_1_/3DPt/HGO/GCE (c), BSA/Ab_1_/3DPt/HGO/GCE(d),CEA/BSA/Ab_1_/3DPt/HGO/GCE(e), and Ab_2_/CEA/BSA/Ab_1_/3DPt/HGO/GCE (f) in 0.10 M KCl containing 2 × 10^−3^ M K_3_[Fe(CN)_6_]/K_4_[Fe(CN)_6_]. Inset is the equivalent circuit.

CV and EIS are common electrochemical methods to examine electrode surface modification. Figure 3A shows the CVs of Fe(CN)_6_^3^^−^/Fe(CN)_6_^4^^−^ at the bare GCE (curve a), 3DPt/HGO/GCE (curve b), Ab_1_/3DPt/HGO/GCE (curve c), BSA/Ab_1_/3DPt/HGO/GCE (curve d), CEA/BSA/Ab_1_/3DPt/HGO/GCE (curve e), and Ab_2_/CEA/BSA/Ab_1_/3DPt/HGO/GCE (curve f), respectively. It can be seen from Figure 3A that the 3DPt/HGO/GCE redox peak current was higher than that of the bare GCE, which means that 3DPt/HGO/GCE showed better charge transfer performance than bare GCE. This was due to the use of the highly porous material 3DPt/HGO, which can increase the electroactive area of the electrode, resulting in a higher redox current. When the electrodes were sequentially modified with Ab_1_, BSA, CEA, and Ab_2_, the peak current gradually decreased, which was attributed to the non-conductivity of Ab_1_, BSA, CEA, and Ab_2_ molecules. The modification of these insulative molecules on the electrode surface hindered the electron transfer, which resulted in a decrease of the redox peak currents.

Figure 3B shows the EIS of the electrode at each assembly step. The impedance spectra include two parts: a semicircle portion and a linear portion. The former represents the electron transfer process and the latter corresponds to the diffusion process. The semicircle diameter equals the electron transfer resistance, R_et_. For the bare GCE, it showed a R_et_ value of 95 Ω. After the electrode was modified with 3DPt/HGO on the surface, a lower resistance (the smaller-diameter semicircle) was shown, which is attributed to the fact that the good conductivity of the 3DPt/HGO has a lower electron transfer resistance than bare GCE. When Ab_1_ (c), BSA (d), CEA (e), and Ab_2_ (f) were adsorbed on the electrode step by step, the R_et_ value increased gradually to 267 Ω, 387 Ω, 506 Ω, and 695 Ω, respectively. This was due to the poor conductivity of these molecules blocking the electron transfer on the redox probe. The results were consistent with previous studies [22,41,42]. These EIS results were consistent with the CV curves shown in Figure 3A. The above experimental results indicate that the immunosensor was successfully fabricated.

We ran a control experiment that involved Ab_2_/Au NPs with and without CEA immobilized onto the electrode surface. In the absence of CEA, a very small signal was shown. When there was 0.1 ng/mL CEA, a high signal was shown. We think this was due to the fact that no sandwich immunoassay was formed without the CEA.

### 3.3. Optimization of Experimental Conditions

**Figure 4:**
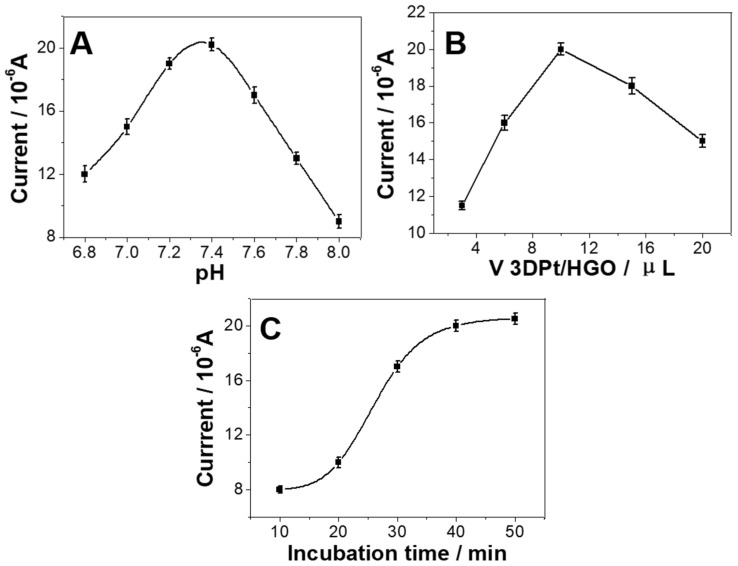
Effect of (**A**) pH, (**B**) the volume of 3DPt/HGO, and (**C**) the incubation time on the differential pulse voltammetry (DPV) response during the detection of CEA.

To obtain the optimal performance of the immunoassay, experimental conditions such as the pH value, the volume of 3DPt/HGO, and the incubation time were studied. The pH value is the most influential factor in these experiments, because an acidic or alkaline solution can affect the antigen-antibody linkage and the activity of biomolecules. Figure 4A shows that when the pH value of PBS changed from 6.8 to 7.4, the current responses of the proposed immunosensor increased. When the pH values of PBS changed from 7.4 to 8.0, the current responses of the proposed immunosensor decreased. The highest current was reached at pH 7.4. This is consistent with the pH of body fluid, meaning that antigens and antibodies will maintain their bioactivity at a near-neutral pH. Therefore, the pH of 7.4 was used for the PBS solution in our further research.

As illustrated in Figure 4B, when 3 μL, 6 μL, and 10 μL of 3DPt/HGO was modified on the electrode, it was found the that the immunosensor had a higher current response when detection of CEA (10 ng/mL) compared with the volume of 3Dpt/HGO used on the electrode of 3 μL or 6 μL. This suggests that more 3DPt/HGO can load a higher amount of Ab_1_. However, when 15 μL/20 μL 3DPt/HGO was modified on the electrode, the peak currents of the immunosensors decreased. This might mean that too much 3DPt/HGO modified on the immunosensor‘s surface will block the electron transfer. Therefore, 10 μL 3DPt/HGO was used for the fabrication of the immunosensors.

The influence of the incubation time on the immunosensor was also investigated. The immunosensor was incubated in CEA solutions for various longer times at 37 °C. Figure 4C shows that longer incubation time led to higher current responses for the detection of CEA. After 40 min, the DPV current tended to not increase any further, indicating that the captured CEA antigen reached saturation. Therefore, 40 min incubation time was chosen for the further experiments [43,44].

### 3.4. Sensor Response Characteristics

**Figure 5:**
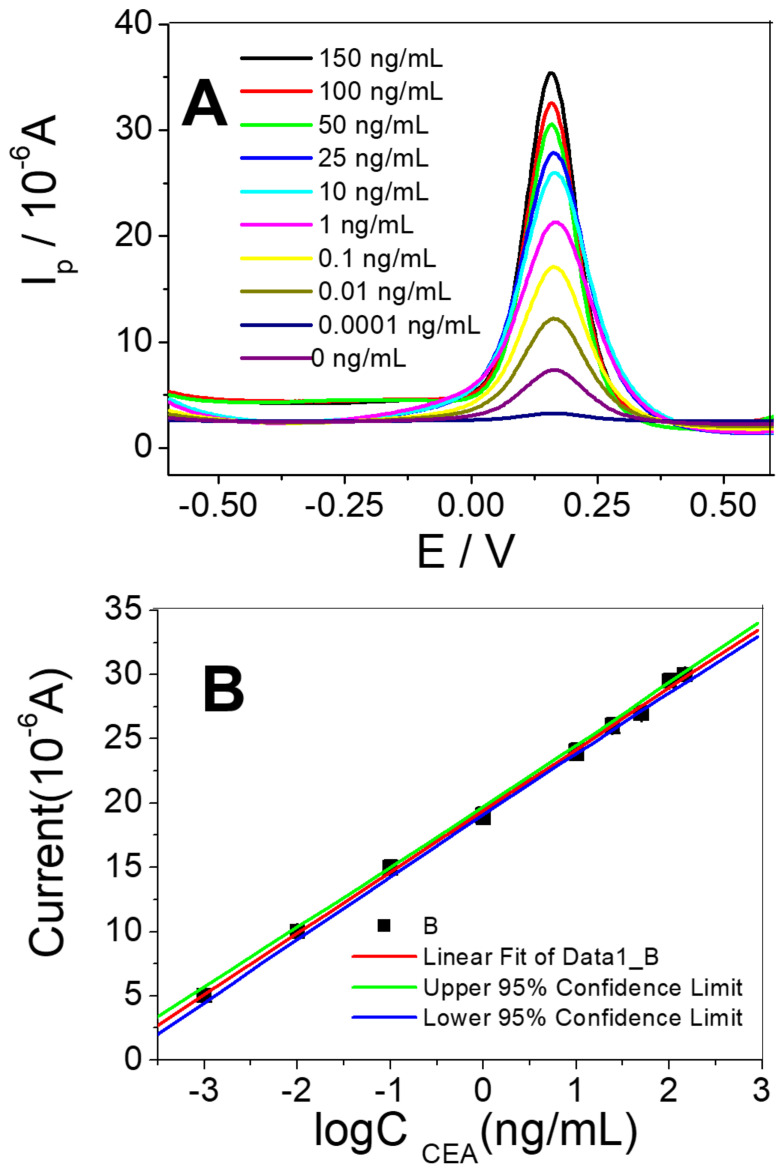
(**A**) DPV response of the immunosensor to different concentrations of CEA (top-down: 150, 100, 50, 25, 10, 1, 0.1, 0.01, 0.001, 0 ng/mL); (**B**) Calibration curve of the immunosensor to different concentrations of CEA.

Under the optimized conditions, different concentrations of CEA were measured by immunosensors, and their DPV signals were recorded. The peak current of the CEA/BSA/Ab_1_/3DPt/HGO can be seen in Figure 5A.

The immunosensor’s response increased gradually with the increase of CEA concentration. The calibration plots (Figure 5B) display a good linear relationship between the peak currents and the log concentration of analytes in the range of 0.001 ng/mL to 150 ng/mL for CEA. The linear response obtained between the peak current (△I) and logC_CEA_ concentration was △I (μA) = 19.4342 + 4.7782 logC_CEA_ (ng/mL). The correlation coefficient was 0.9993 and the limit of detection was 0.0006 ng/mL. Based on these results, it is concluded that the proposed immunosensor achieved a satisfactory detection limit and had a logarithmic linear response. Compared with most of the sensors previously developed for CEA detection, as summarized in Table 1, this immunosensor exhibited a similar detection limit and a linear range.

### 3.5. Selectivity, Reproducibility, and Stability of Immunosensors

**Figure 6:**
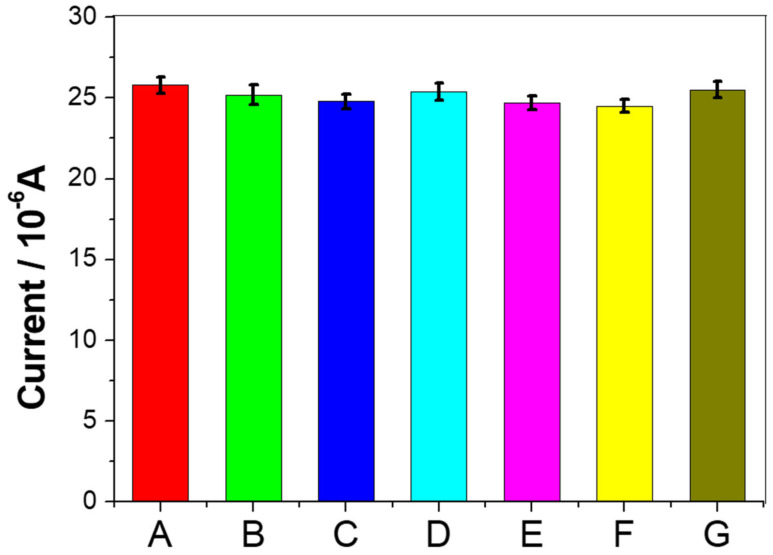
Study of the specificity of the immunosensor towards various interfering compounds. (A) 10 ng/mL CEA; (B) 10 ng/mL CEA + 100 ng/mL L-cysteine; (C) 10 ng/mL CEA + 100 ng/mL AFP; (D) 10 ng/mL CEA + 100 ng/mL glucose; (E) 10 ng/mL CEA + 100 ng/mL L-glutamine; (F) 10 ng/mL CEA + 100 ng/mL vitamin C; (G) 10 ng/mL CEA + 100 ng/mL human serum. Error bars represent the standard deviation, *n* = 5.

In order to evaluate the selectivity of the immunosensor, several interfering compounds that may exist in the human environment were introduced, including L-cysteine, AFP, glucose, L-glutamine, vitamin C, and human serum. This immunosensor was incubated with 10 ng/mL CEA containing one of the above-mentioned compounds (100 ng/mL), respectively, as shown in Figure 6, where A is pure CEA without interference, and B, C, D, E, F, and G are mixtures of CEA and L-cysteine, AFP, glucose, L-glutamine, vitamin C, and human serum, respectively. The change in current caused by the interfering compound was less than 5.4% compared to no interference, indicating that the immunosensor had good selectivity.

Reproducibility is an important indicator for evaluating the performance of immunosensors. Five immunosensors were repeatedly prepared by the same method for measuring 10 ng/mL CEA. The relative standard deviation of the peak current obtained was 5.1%, indicating that the sensor had good reproducibility. In addition, the stability of the immunosensor was studied by keeping the immunosensor in the PBS solution (pH 7.4) at 4 °C for 2 weeks. It was found that the immunosensor could retain 96% of the original electrochemical signal strength. The results indicate that the immunosensor had good stability.

### 3.6. Real Sample Analyses

In order to evaluate the practical application of the proposed immunosensor in the detection of CEA in biological samples, the standard addition method was applied. For this purpose, 30 µL of human serum was diluted to 3.0 mL with PBS. Firstly, different amounts of CEA were added to the 1 mL vessel, then the above diluted samples were added for further detection. Table 2 shows the analytical results and recoveries. The recoveries of the spiked samples varied in the range of 94.20–107%. The relative standard deviation (RSD) was obtained in the range of 1.62–2.86%. It was clear that the fabricated immunosensor had significant potential for detecting CEA in real serum samples.

## 4. Conclusions

In this study, a new type of CEA electrochemical immunosensor was constructed using Pt nanoparticles and HGO. The sensing interface of the sensor combined the advantages of nanoplatinum and porous graphene, and had a large specific surface area, excellent electrical conductivity, biocompatibility, and good catalysis, which not only greatly increased the fixed amount of biomolecules, but also significantly improved sensor sensitivity and stability. The electrochemical immunosensor based on 3DPt/HGO composites was used to detect CEA, with a wide response range of 0.001 ng/mL to 150 ng/mL and a lower detection limit of 0.0006 ng/mL, which provides a new method for the detection of CEA. The immunosensor has the advantages of simple manufacture, convenient use, low cost, and has potential application value in biomedicine, clinical diagnosis, health detection, and more.

## Figures and Tables

**Table 1:** Comparison of the response characteristics of different modified electrodes.

Electrode	Detection Range	Detection Limit	Method	Ref.
Pt/ionic liquid/graphene	0.001 fg/mL−1 ng/mL	0.0003 fg/mL	ECL	[32]
Nitrogen-doped graphene/PtPd	5 fg/mL–50 ng/mL	2 fg/mL	A.C. impedance	[33]
NiAuPt nanoparticles on graphene	0.001–100 ng/mL	0.27 pg/mL	DPV	[22]
Platinum nanoparticles /PdPt nanocages	0.05–200 ng/mL	1.4 pg/mL	A.C. impedance	[34]
Graphene/platinum nanoparticles	0.01–100 ng/mL	1.64 pg/mL	DPV	[35]
3DPt/HGO	0.001–150 ng/mL	0.0006 ng/mL	DPV	this work

**Table 2:** Detection of CEA in human serum samples.

Samples	Added (ng/mL)	Found (ng/mL)	Recovery (%)	RSD (%)
1	0.10	0.107	107.00	2.86
2	1.00	0.942	94.20	2.33
3	10.00	10.480	104.80	1.62
